# The Efficacy of Metal Artifact Reduction Mode in Cone-Beam Computed Tomography Images on Diagnostic Accuracy of Root Fractures in Teeth with Intracanal Posts

**DOI:** 10.22037/iej.v13i1.17352

**Published:** 2018

**Authors:** Zahra Dalili Kajan, Mehran Taramsari, Negar Khosravi Fard, Farnoosh Khaksari, Forough Moghasem Hamidi

**Affiliations:** a *Department of Maxillofacial Radiology, Dental School, Guilan University of Medical Sciences, Rasht, Iran; *; b * Department of Endodontics, Dental School, Guilan University of Medical Sciences, Rasht, Iran; *; c * Dental School, Guilan University of Medical Sciences, Rasht, Iran*

**Keywords:** Artifact, Cone-Beam Computed Tomography, Diagnosis, Tooth Fracture, Vertical Root Fracture

## Abstract

**Introduction:**

The purpose of this study was to evaluate the efficacy of cone-beam computed tomography CBCT in the diagnosis of RF in the presence of an intracanal posts with and without applying “metal artifact reduction” (MAR) mode.

**Methods and Materials:**

This *in vitro* study included 60 single-canal endodontically treated premolars. Post spaces were created in all roots. RFs were simulated in 30 of the 60 teeth. Dentatus posts were cemented in 15 of 30 roots with and without RFs. Teeth were arranged randomly in 6 artificial dental arches. Images were taken using a Vatech CBCT machine with and without MAR (MAR and WMAR, respectively). A radiologist and an endodontist evaluated the CBCT images for the presence of RFs. Sensitivity, Specificity, positive and negative predictive values were determined for each mode. MC Nemar’s and Kappa tests were used for data analysis.

**Results:**

The percentage of correct diagnosis using the WMAR mode in both the post space and pin groups in the presence of root fracture was 46.6%; with MAR, it increased to 86.6% and 66.6%, respectively. There was no significant difference between two modes in post space (*P*=0.503) and metal pin groups (0.549). The overall sensitivity of VRF diagnosis in WMAR mode was 46.67%; in MAR mode, sensitivity was 76.67%. The specificity of WMAR and MAR modes were 60% and 53.33%. The levels of agreement between two modes and real findings were less than 0.45.

**Conclusions:**

There were no significant differences between the efficacies of imaging modes. The sensitivity of the MAR mode for diagnosis of VRF in both the pin and post space groups was higher than the WMAR mode. The specificity of MAR in comparison with WMAR was less or equal in dental groups. The agreement between CBCT and real findings was poor.

## Introduction

Vertical root fracture (VRF) is one of the most common reasons for extraction of an endodontically treated tooth [1]. A major cause of RF is insertion of posts into endodontically treated teeth, leading to considerable structural loss [2]. Iatrogenic causes of VRF include high wedging forces following lateral condensation of gutta-percha, over-preparation of the canal for insertion of the dental post, choosing an inappropriate size of intra-canal post, and traumatic placement of intra-canal restorations [3, 4]. To prevent damage to the periodontium and choose the appropriate treatment, early diagnosis of tooth root fracture is important [5]. Detection of RF is complicated, and clinicians must use both clinical and radiographic evidence for diagnosis [2].

**Figure 1:**
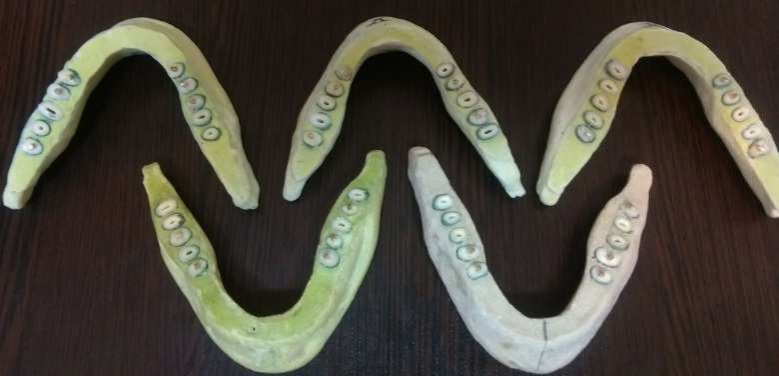
Arches with the same forms

Cone-beam computed tomography (CBCT) offers sub-millimeter resolution, short scanning times, low radiation dose, and three-dimensional (3D) illustrations that are reasons CBCT imaging is being widely used in dental diagnostics [6, 7]. Several investigations have evaluated the efficacy and value of CBCT in RF diagnosis [8, 9]. CBCT images had higher sensitivity and diagnostic efficiency for the detection of root fractures compared to intraoral radiography and multi-detector helical CT [10]. The CBCT method also has a high accuracy of VRF diagnosis [2, 11, 12].

In CBCT imaging, the photons of the x-ray beam include a different spectrum of photon energy. When the x-ray beam passes through an object, lower-energy photons are absorbed. This results in beam hardening effects in the path of highly attenuating objects such as metal structures, which present as a metal artifact [13-15]. This artifact can negatively influence the CBCT image quality by reducing the image contrast and obscuring structures [16, 17]. Attempts to reduce these artifacts are metal artifact reduction (MAR) [14].

As mentioned, CBCT has been a reliable method for the detection of VRF. However, the presence of metal intracanal posts and gutta-percha can interfere with the diagnostic capability of CBCT images and decrease the sensitivity, specificity and accuracy of VRF detection [12, 18, 19].

Some CBCT machines, such as Pax-i3D (Vatech, Gyeonggi-do, Korea), have an option for MAR mode. Application of MAR to CBCT images could effectively reduce image noise when a phantom had a metal object [20, 21]. Application of MAR did not increase the diagnostic value of CBCT for detection of RF, but there are limited documents to confirm this finding [16, 22]. Thus, the purpose of this study was to evaluate the value of CBCT in RF diagnosis in endodontically treated teeth containing an intracanal post, with and without application of the MAR mode.

**Figure 2:**
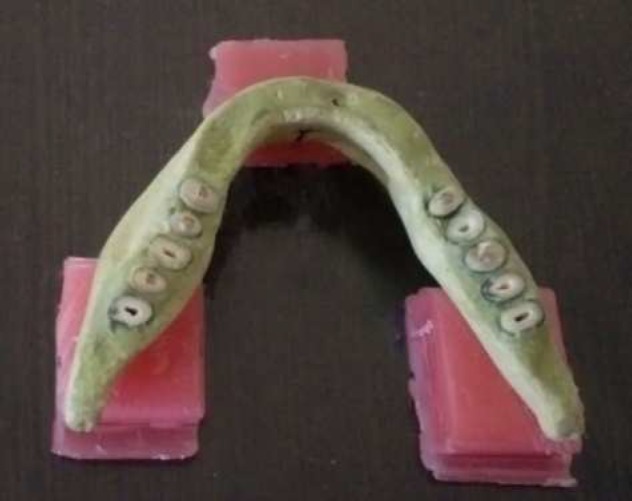
A fixed mandible model on a plexiglass sheet

## Materials and Methods

This *in vitro* study was performed on 60 human single-canal premolar teeth. All teeth were free from decay, fractures, or cracks. The ethic approval number for this study was IR.GUMS.REC.1394.448. Absence or presence of root fracture on root surfaces are confirmed by endodontist with direct observation by using magnifier and in enough light condition. They also did not have severe root curvature. The other exclusion criteria were open apex, internal resorption and previous root canal treatment. To select the teeth, right angle periapical radiography with 30-degree horizontal angulation was taken of all selected teeth using phosphor plate (PSP) sensors (Digora, Soredex, Helsinki, Finland) to confirm a non-calcified single root canal. This finding was confirmed by an expert endodontist. The root surfaces of the selected teeth were cleaned of any calculus or tissue. Teeth were placed in 5.25% hypochlorite solution for 24 h. Then, the crowns were cut from the cementoenamel junction (CEJ) with a diamond disk and the root canal treatments (RCTs) were performed. Preparation was done using the step back method with a K-file to master apical file which was set at #40. Flaring was performed using #2 and 3 Gates Gliden drills (Dentsply Maillefer, Ballaigues, Switzerland) to 1/2 to 2/3 of the working length. The canals were obturated using the cold lateral condensation technique with gutta-percha and AH-26 sealer (Dentsply Maillefer, Ballaigues, Switzerland).

After 24 h, post spaces were prepared using #3 and #4 Gates Gliden drills to 1/2 of the working length. The remaining gutta-percha in the canal was condensed with a plugger. Then, crack lines were created in 30 teeth. To create crack lines, a post larger than the provided space was placed in the root canal and turned by wrench to create a crack. In this study, we attempted to create fine crack lines without displacement of broken pieces. The crack lines had to be visible for the naked eye. If the crack or fracture line were not vertically or longitudinally along root surface they were excluded from the study and new tooth was added. To simulate the periodontal ligament (PDL) space, the surface of the roots was covered with a 0.5-mm layer of green wax.

**Figure3:**
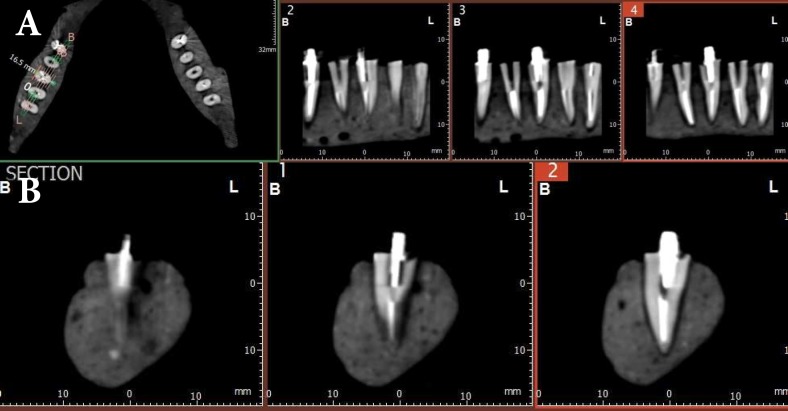
A, B) A fracture line on the right third tooth from the end of dental arch in mesiodistal cross-sectional CBCT images after applying metal artifact reduction (MAR)

In 30 teeth with cracked and non-cracked roots, metal pins (Dentatus, Stockholm, Sweden) were cemented into the canal using a self-curing glass ionomer.

The tooth roots were divided into 4 groups of 15 teeth. Teeth in group 1 had crack lines, and Dentatus posts were cemented into the roots. Group 2; teeth had crack lines, and the post spaces were left empty. Group 3; teeth had no crack lines and Dentatus posts were cemented into the roots. Group 4; teeth had no crack lines, and the post spaces were left empty.

An alginate impression was taken from an edentate mandible model. A mixture of immediate acryl, stone plaster, and sawdust in a proportion of 2:2:1 was poured into the mold. The teeth were set in 6 artificial arches; each arch contained 10 teeth (Figure 1). The teeth from 4 groups in 6 tooth arches were randomly presented to blind the observers. 

The 6 prepared arches were fixed on plexiglass acrylic sheets with their occlusal planes parallel to the ground (Figure 2). Finally, the fixed arches were transferred to the "Pax-i3D" CBCT apparatus (Vatech Co., Ltd., Gyeonggi-do, Korea(.

In "EzDent-i" software (Vatech Co., Ltd. Gyeonggi-do, Korea), a CT program was selected and exposure settings were adjusted as follows: 90×120 mm field of view (FOV), vertical position on mandible, horizontal position in center, image quality item on low dose, a 0.2-mm standard voxel size and male icon as gender. The imaging of each arch was performed once with and once without the MAR mode. The exposure factors were 95 kVp and 6 mA and 5.9 sec exposure time.

A maxillofacial radiologist and an endodontist both with more than 15 years of experiences were involved in different RF studies; they observed the CBCT images in "Ez 3D Plus" software (Vatech Co., Ltd., Hwaseong, Korea) to determine the presence or absence of a fracture using axial (thickness of 1mm and a distance of 1mm) and cross-sectional images (thickness of 1 mm and a distance of 0.5 mm). They were allowed to use all facilities of image processing in the software, but were blinded to the use of MAR or WMAR modalities. We used expert observers that had been involved in previous studies of RF as researchers and observes to reduce the variability of observers and focus on the effects of applying these modes.

The observers ranked their findings as follows: grade 1: definitely absence of fracture; grade 2: probably absence of fracture; grade 3: probably presence of fracture; and grade 4: definitely presence of fracture.

Figure 3 depicts a tooth classified as reveals “definitely presence of RF” as grade 4 in third tooth from end of dental arch.

McNemar’s test was used to compare the percentage of correct diagnosis in each CBCT mode. The agreement between CBCT and gold standard in MAR and WMAR modes were determined by kappa agreement test. Sensitivity, specificity, positive predictive value (PPV), and negative predictive value (NPV) were calculated for both modes by comparing observers' data with a gold standard.

## Results

In this *in vitro* study, 60 single-canal premolar teeth with VRF (*n*=30) and without VRF (*n*=30) were used to evaluate the ability of CBCT to detect VRF with the presence of an intracanal pin with and without using the MAR mode. Based on Kappa analysis, the observers had 85% agreement on images taken in WMAR mode and 87% on images taken using the MAR mode.

The percentage of correct diagnosis of the presence of RF in both groups (metal pin and post space) was 46.6% in the WMAR mode and 76.6% in the MAR mode. This percentage in the MAR mode for teeth with a metal pin in the canal was 66.6%; absence of the metal pin increased the frequency of correct diagnosis to 86.6% (Table 1). Therefore, the highest level of the correct diagnosis of the presence of RF was observed in the post space group by applying the MAR mode. 

In the evaluation of 60 teeth in WMAR mode, the 2 observers detected the presence or absence of RF correctly in 53.3% of teeth. Among the correct diagnosis, 6% of observers’ reports were “probably negative” (3% in the pin group and 3% in the post space group), and 12% of their correct reports were “probably positive” (9% in the pin group and 3% in the post space group). In MAR mode, the correct reports of the observers were 65%; only 2.5% of teeth in the post space group were considered “probably positive”.

**Table 1:** The frequency of correct diagnosis of root fractures using MAR and WMAR modes

**WMAR Mode** ^b^	**MAR Mode** ^a^	**Number of teeth with RF**	
46.6%	86.6%	15	**Post space **
46.6%	66.6%	15	**Metal pin **
46.6%	76.6%	30	**Total **

a MAR: Metal artifact reduction;

b WMAR: Without metal artifact reduction

**Table 2:** The frequency of the correct diagnosis in both imaging modes in two dental groups

***P*** **-value** ^c^	**WMAR mode** ^b^	**MAR mode** ^a^	**The number of evaluated teeth**	
0.503	50%	63.3%	30	**Post space**
0.549	56.6%	66.7%	30	**Metal pin**
0.281	53.3%	65%	60	**Total**

a MAR: Metal artifact reduction;

c McNemar test

**Table 3:** Diagnostic values of MAR and WMAR modes in RF diagnosis

	**Dental groups**					
	**Post space**		**Metal pin**		**Total**	
**CBCT Mode **	WMAR ^a^	MAR ^b^	WMAR	MAR	WMAR	MAR
**Kappa Value**	0.0	0.27	0.13	0.33	0.07	0.3
**Agreement**	poor	poor	poor	poor	poor	poor

a WMAR : Without metal artifact reduction;

b MAR: Metal artifact reduction

**Table 4:** Diagnostic values of MAR and WMAR modes in RF diagnosis

	**Post space **		**Metal pin **		**Total **	
	**WMAR** **	**MAR** *	**WMAR **	**MAR **	**WMAR **	**MAR **
**Sensitivity** ** %** ** (CI) **	46.67 (22.28-72.58)	86.67 (58.39-97.66)	46.67 (22.28-72.85)	66.67 (38.69-87.01)	46.67 (28.80-65.36)	76.67 (57.30-89.36)
**Specificity** ** %** ** (CI) **	53.33 (27.42-77.72)	40 (17.46-67.1)	66.67 (38.69-87.01)	66.67 (38.69-87.01)	60 (40.75-76.78)	53.33 (34.64-71.20)
**(PPV)** ** % ** **(CI)**	50 (24.04-75.96)	59.09 (36.68-78.52)	58.33 (28.60-83.50)	66.67 (38.69-87.01)	53.85 (33.75-72.86)	62.16 (44.79-77.06)
**(NPV) % (CI) **	50 (25.51-74.49)	75 (35.58-95.55)	55.56 (31.35-77.60)	66.67 (38.69-87.01)	52.94 (35.40-69.86)	69.56 (46.99-85.94)
**False Negative %**	26.7	6.7	26.6	16.6	53.3	23.3
**False positive %**	23.4	30.	16.6	16.6	40.0	46.6

CI: confidence interval, PPV: positive predictive value, NPV: negative predictive value

a MAR: Metal artifact reduction;

b WMAR: Without metal artifact reduction

Table 2 reveals the percentage of total correct diagnosis (the true positive and true negative) for both imaging modes in both examined groups. Values were compared using McNemar’s test; a significant difference between the diagnosis values of the 2 imaging modes in the post space group (*P*=0.503), metal pin group (*P*=0.549), and totally (*P*=0.281) for diagnosis of RF was not observed. However, as is seen in the table, the percentage of correct diagnosis, including presence or absence of fracture, was higher in MAR mode than in WMAR mode.

The agreement of CBCT and gold standard in each mode and in the post space and metal pin groups was shown in Table 3. The strength of agreement is considered to be “poor” (less than 0.45). Sensitivity, specificity, PPV, NPV, and false and negative values for diagnosis of RF using both imaging modes for all teeth, metal posts, post space groups are shown in Table 4. 

The total sensitivity of the WMAR mode was 46.67%; this was the same for teeth with or without a metal post in the canal. The total sensitivity of the MAR mode in the presence of RF was 76.67%, and it was higher in the post space group than the metal pin group. By applying MAR mode, the sensitivity in both post space (86.67%) and metal pin (66.67%) groups was more than WMAR mode. In metal pin group specificity was same in MAR and WMAR modes (66.67%) but in post space groups the specificity is decreased in MAR (40%) and WMAR (53.33%) modes. PPV has more percentage in metal pin (66.67%) and post space (59.09%) groups by applying MAR mode. Overall, use of the MAR mode improved RF diagnosis. The improvement observed in the post space group was higher than in the metal pin group.

## Discussion

A difficulty in dentistry is the correct diagnosis of VRF. Clinical and conventional radiographic findings may be helpful for RF diagnosis, but may not be enough, as the RF may appear as an endodontic failure or periodontal lesion. Misdiagnosis leads to unnecessary and aggressive treatment [23]. CBCT images provide a 3D view and are intended to be a helpful tool. However, the metal artifacts make diagnosis difficult. Several studies showed that the presence of metal posts in treated roots have a negative impact on VRF detection due to metal artifact formation [24, 25].

In the present study, the diagnostic values of MAR and WMAR in RF diagnosis were compared. According to the results of this study, when using MAR, the highest percentage of correct diagnosis was reported in the post space group. The diagnostic accuracy percentage of correct diagnosis was equal between the pin and post space groups without using MAR. This finding could be due to the less f negative effects of streak artifacts due to intracanal filling material than the metal pin thus applying the MAR was effective in improving the image quality.

The overall sensitivity of CBCT images was higher when using MAR than when using the WMAR mode (76.67% *vs.* 46.67%), but the rate of specificity reported in MAR mode was lower than when using the WMAR mode (53.33% *vs.* 60%). These results contradict Bechara *et al.* [16] and Bezerra *et al.* (21). This difference may be related to the position and form of used dental arch in relation to field of view (FOV). Queiroz *et al.* [26] showed the effect of MAR in different part of FOV are not equal. It needs more research in clinical diagnosis. Bechara *et al.* [16] studied the effectiveness of artifact reduction (AR) algorithms on the diagnostic accuracy of Master 3D and Pro Max devices. The highest accuracy and sensitivity were obtained using Pro Max without artifact reduction (AR); the lowest accuracy was obtained using the Master 3D with AR. They found that the specificity of the Pro Max device without the AR algorithm was significantly higher. No significant differences were detected when applying AR algorithms using the Master 3D device in comparison with a standard situation. Without the AR algorithm, the specificity of the Pro Max device was higher than that of the Master 3D device. There was no difference between the two devices using the AR algorithm. Bechara *et al.* [16] found that CNR (contrast-to-noise ratio) is one of the qualitative factors of images that improved when using the AR algorithm in Master 3D devices.[16] and Bezerra *et al.* [27]. This difference may be related to the position and form of used dental arch in relation to field of view (FOV). Queiroz *et al.* [26] showed the effect of MAR in different part of FOV are not equal. It needs more research in clinical diagnosis. Bechara *et al.* [16] studied the effectiveness of artifact reduction (AR) algorithms on the diagnostic accuracy of Master 3D and Pro Max devices. The highest accuracy and sensitivity were obtained using Pro Max without artifact reduction (AR); the lowest accuracy was obtained using the Master 3D with AR. They found that the specificity of the Pro Max device without the AR algorithm was significantly higher. No significant differences were detected when applying AR algorithms using the Master 3D device in comparison with a standard situation. Without the AR algorithm, the specificity of the Pro Max device was higher than that of the Master 3D device. There was no difference between the two devices using the AR algorithm. Bechara *et al.* [16] found that CNR (contrast-to-noise ratio) is one of the qualitative factors of images that improved when using the AR algorithm in Master 3D devices. In the present study, this quality may be effective in the improvement in the percentage of correct diagnostic accuracy of RF and increasing the sensitivity.

The AR algorithm in the Bezerra *et al.* [22] study decreased artifact formation but did not have a positive impact on diagnosis of VRFs in root canals with intracanal metal posts. 

The positive and negative predictive values are the indices that reflect the confidence of CBCT when reporting the true positive and negative results. They have been found in MAR mode rather than WMAR mode in our study. 

De Menzes *et al.* [28] evaluated the effects of gutta-percha and metal posts on the diagnostic accuracy of CBCT. They observed that the sensitivities of the three observers in the gutta-percha group were 62.5%, 62.5% and 75%; the sensitivities of the metal post group were 75%, 75% and 62.5%, respectively. Their reported sensitivities were closer to the sensitivity of the MAR mode for both the metal post and post space groups. Unlike the study of De Menzes *et al.* [28], the highest false positive rate of CBCT when using the MAR mode occurred in the post space group. The highest false negative rate was found in WMAR in both post space and metal post groups. It might be related to masking of RF by streak artifacts of gutta-percha or metal pin.

Hassan *et al.* [29] revealed that the sensitivity and diagnostic accuracy of the CBCT scanners was significantly higher than in periapical images in RF detection. The overall diagnostic accuracy of root fracture in the CBCT scanner was higher (0.86) than the accuracy of periapical images (0.66). The canal filling materials did not reduce CBCT scanner accuracy, but the presence of gutta-percha greatly reduced CBCT specificity [29].

In the present study, the use of metal posts in relation to post space groups reduced sensitivity from 86.6% to 66.6%; application of the MAR algorithm increased specificity from 40% to 66.6%. In WMAR mode, in the metal post and post space groups, the level of sensitivity was unchanged but specificity increased. Also in the MAR mode, the presence of the metal post increased the positive predictive value (PPV) and decreased the negative predictive value (NPV) compared with the post space group. Hassan *et al.* [30] confirmed the effect of root canal filling materials in five CBCT devices in RF diagnosis. They found that the root canal filling materials did not change the sensitivity of CBCT scanners, but reduced the specificity of CBCT in RF line diagnosis. It suggests that the root canal filling materials did not affect the accuracy of fracture line detection, but reduced the accuracy of healthy tooth diagnosis.

Other studies showed that a smaller voxel size increased the accuracy of CBCT in the diagnosis of VRF [5, 31-33]. In this study, other important aspects of increasing accuracy were application of the MAR mode and the expertise of the observers. To decrease the variable of observers’ skills in these types of studies, we considered the observers experiences in the field.

In addition, the sensitivity in the post space group was equal to or greater than the metal post group in both modes. This finding is similar to the findings of Hassan *et al.* [29] and Wang *et al.* [29] and Wang *et al.* [34] that showed root canal filling materials reduced CBCT sensitivity. They concluded that streak artifacts originating from gutta-percha could be similar to a fracture line and reduce CBCT image sensitivity.Taramsari *et al.* [35] reported that the HiRes mode of CBCT for RF diagnosis was more accurate than standard modes. In the present study, the use of the MAR mode in both metal pin and post space groups increased the accuracy of the RF diagnosis.

## Conclusion

The correct diagnosis of RF by applying MAR in CBCT was increased in both the metal pin and post space groups, but there was no significant difference between correct diagnosis in MAR and WMAR modes. 

The sensitivity of MAR was higher than WMAR for RF diagnosis. However, specificity was not improved by applying MAR. The false positive results due to application of MAR must be considered when interpreting RFs.

## References

[1] Khasnis SA, Kidiyoor KH, Patil AB, Kenganal SB (2014). Vertical root fractures and their management. J Conserv Dent.

[2] Dhawan A GS, Mittal R (2014). Vertical root fractures: An update review. JResDent.

[3] Tamse A (2016). Vertical root fractures in endodontically treated teeth:diagnostic signs and clinical management. Endodontic Topics.

[4] Jakobson SJ, Westphalen VP, Silva Neto UX, Fariniuk LF, Schroeder AG, Carneiro E (2014). The influence of metallic posts in the detection of vertical root fractures using different imaging examinations. Dentomaxillofac Radiol.

[5] Kamburoglu K, Murat S, Yuksel SP, Cebeci AR, Horasan S (2010). Detection of vertical root fracture using cone-beam computerized tomography: an in vitro assessment. Oral Surg Oral Med Oral Pathol Oral Radiol Endod.

[6] Scarfe WC, Farman AG, Sukovic P (2006). Clinical applications of cone-beam computed tomography in dental practice. J Can Dent Assoc.

[7] Kiarudi AH, Eghbal MJ, Safi Y, Aghdasi MM, Fazlyab M (2015). The applications of cone-beam computed tomography in endodontics: a review of literature. Iran Endod J.

[8] Ma RH, Ge ZP, Li G (2016). Detection accuracy of root fractures in cone-beam computed tomography images: a systematic review and meta-analysis. Int Endod J.

[9] Kajan ZD, Taromsari M (2012). Value of cone beam CT in detection of dental root fractures. Dentomaxillofac Radiol.

[10] Iikubo M, Kobayashi K, Mishima A, Shimoda S, Daimaruya T, Igarashi C, Imanaka M, Yuasa M, Sakamoto M, Sasano T (2009). Accuracy of intraoral radiography, multidetector helical CT, and limited cone-beam CT for the detection of horizontal tooth root fracture. Oral Surg Oral Med Oral Pathol Oral Radiol Endod.

[11] Moudi E, Haghanifar S, Madani Z, Alhavaz A, Bijani A, Bagheri M (2014). Assessment of vertical root fracture using cone-beam computed tomography. Imaging Sci Dent.

[12] Valizadeh S, Vasegh Z, Rezapanah S, Safi Y, Khaeazifard MJ (2015). Effect of Object Position in Cone Beam Computed Tomography Field of View for Detection of Root Fractures in Teeth with Intra-Canal Posts. Iran J Radiol.

[13] Draenert FG, Coppenrath E, Herzog P, Muller S, Mueller-Lisse UG (2007). Beam hardening artefacts occur in dental implant scans with the NewTom cone beam CT but not with the dental 4-row multidetector CT. Dentomaxillofac Radiol.

[14] Boas FE, Fleischmann D (2012). CT artifacts: Causes and reduction techniques. Imaging Med.

[15] white SC, Pharoah MJ (2014). Oral radiology principles and inrepretation.

[16] Bechara B, Alex McMahan C, Moore WS, Noujeim M, Teixeira FB, Geha H (2013). Cone beam CT scans with and without artefact reduction in root fracture detection of endodontically treated teeth. Dentomaxillofac Radiol.

[17] Schulze R, Heil U, Gross D, Bruellmann DD, Dranischnikow E, Schwanecke U, Schoemer E (2011). Artefacts in CBCT: a review. Dentomaxillofac Radiol.

[18] Neves FS, Freitas DQ, Campos PS, Ekestubbe A, Lofthag-Hansen S (2014). Evaluation of cone-beam computed tomography in the diagnosis of vertical root fractures: the influence of imaging modes and root canal materials. J Endod.

[19] Mohammadpour M, Bakhshalian N, Shahab S, Sadeghi S, Ataee M, Sarikhani S (2014). Effect of titanium and stainless steel posts in detection of vertical root fractures using NewTom VG cone beam computed tomography system. Imaging Sci Dent.

[20] Bechara B, McMahan CA, Geha H, Noujeim M (2012). Evaluation of a cone beam CT artefact reduction algorithm. Dentomaxillofac Radiol.

[21] Bechara BB, Moore WS, McMahan CA, Noujeim M (2012). Metal artefact reduction with cone beam CT: an in vitro study. Dentomaxillofac Radiol.

[22] Bezerra IS, Neves FS, Vasconcelos TV, Ambrosano GM, Freitas DQ (2015). Influence of the artefact reduction algorithm of Picasso Trio CBCT system on the diagnosis of vertical root fractures in teeth with metal posts. Dentomaxillofac Radiol.

[23] Safi Y, Aghdasi MM, Ezoddini-Ardakani F, Beiraghi S, Vasegh Z (2015). Effect of Metal Artifacts on Detection of Vertical Root Fractures Using Two Cone Beam Computed Tomography Systems. Iran Endod J.

[24] Ozer SY (2010). Detection of vertical root fractures of different thicknesses in endodontically enlarged teeth by cone beam computed tomography versus digital radiography. J Endod.

[25] da Silveira PF, Vizzotto MB, Liedke GS, da Silveira HL, Montagner F, da Silveira HE (2013). Detection of vertical root fractures by conventional radiographic examination and cone beam computed tomography - an in vitro analysis. Dent Traumatol.

[26] Queiroz PM, Santaella GM, Paz TDJd, Freitas DQ (2017). Evaluation of a metal artefact reduction tool on different positions of a metal object in the FOV. Dentomaxillofacial Radiology.

[27] Abazarpour R, Parirokh M, Farhadi A, Jalali Z, Kheirabadi N (2017). Successful Ultra-Conservative Management of a Mandibular Premolar with Dens Invaginatus. Iran Endod J.

[28] Menezes RF, Araujo NC, Santa Rosa JM, Carneiro VS, Santos Neto AP, Costa V, Moreno LM, Miranda JM, de Albuquerque DS, Albuquerque M, Dos Santos RA, Gerbi ME (2016). Detection of vertical root fractures in endodontically treated teeth in the absence and in the presence of metal post by cone-beam computed tomography. BMC Oral Health.

[29] Hassan B, Metska ME, Ozok AR, van der Stelt P, Wesselink PR (2009). Detection of vertical root fractures in endodontically treated teeth by a cone beam computed tomography scan. J Endod.

[30] Hassan B, Metska ME, Ozok AR, van der Stelt P, Wesselink PR (2010). Comparison of five cone beam computed tomography systems for the detection of vertical root fractures. J Endod.

[31] Wenzel A, Haiter-Neto F, Frydenberg M, Kirkevang LL (2009). Variable-resolution cone-beam computerized tomography with enhancement filtration compared with intraoral photostimulable phosphor radiography in detection of transverse root fractures in an in vitro model. Oral Surg Oral Med Oral Pathol Oral Radiol Endod.

[32] Edlund M, Nair MK, Nair UP (2011). Detection of vertical root fractures by using cone-beam computed tomography: a clinical study. J Endod.

[33] Patel S, Brady E, Wilson R, Brown J, Mannocci F (2013). The detection of vertical root fractures in root filled teeth with periapical radiographs and CBCT scans. Int Endod J.

[34] Wang P, Yan XB, Lui DG, Zhang WL, Zhang Y, Ma XC (2011). Detection of dental root fractures by using cone-beam computed tomography. Dentomaxillofac Radiol.

[35] Taramsari M, Kajan ZD, Bashirzadeh P, Salamat F (2013). Comparison of high-resolution and standard zoom imaging modes in cone beam computed tomography for detection of longitudinal root fracture: An in vitro study. Imaging Sci Dent.

